# Melatonin receptor signaling in human pathologies: from molecular mechanisms to therapeutic targets

**DOI:** 10.1152/function.011.2026

**Published:** 2026-05-15

**Authors:** Yen-Sung Huang, Sheng-Ding Wu, Hsi-Chih Chen, Kuo-Cheng Lu, Shuk-Man Ka, Chia-Chao Wu

**Affiliations:** ^1^Institute of Biomedical Sciences, Academia Sinica, Taipei, Taiwan; ^2^Graduate Institute of Aerospace and Undersea Medicine, National Defense Medical University, Taipei, Taiwan; ^3^School of Medicine, National Yang Ming Chiao Tung University, Taipei, Taiwan; ^4^Division of Nephrology, Department of Internal Medicine, Tri-Service General Hospital, National Defense Medical University, Taipei, Taiwan; ^5^Division of Nephrology, Department of Medicine, Taipei Tzu Chi Hospital, Buddhist Tzu Chi Medical Foundation, New Taipei City, Taiwan; ^6^Department and Graduate Institute of Microbiology and Immunology, National Defense Medical University, Taipei, Taiwan

**Keywords:** melatonin receptor, MTNR1A, MTNR1B, signal transduction, single nucleotide polymorphism

## Abstract

The melatonin receptor 1 A (MTNR1A), a highly conserved G protein-coupled receptor (GPCR), mediates crucial physiological functions. Its structure features an N-terminus responsible for melatonin binding and a C-terminus that initiates downstream signaling pathways to modulate target gene expression. Given MTNR1A’s ability to form homodimers or heterodimers with other GPCRs, its expression levels are critical for the precise control of cellular signaling. This review article provides a comprehensive update on MTNR1A, highlighting recent developments concerning its expression distribution, gene regulation, protein motifs, and mediated signaling pathways. We also discuss the clinical relevance of single-nucleotide polymorphisms (SNPs) associated with the MTNR1A receptor and the range of diseases linked to its dysfunction. Current understanding and future perspectives regarding gene regulation and the stimulation of MTNR1A expression are critically addressed. Furthermore, we investigate the role of MTNR1A genetic variants in idiopathic osteoporosis and the association between decreased MTNR1A expression and membranous nephropathy. The systemic involvement of MTNR1A downregulation in cancer, fetal growth restriction, type 2 diabetes, and Parkinson’s and Alzheimer’s diseases is further underlined by its established biological functions. In conclusion, targeting MTNR1A-related downregulation and developing specific agonists or modulators offer a promising avenue for advancement in therapeutic medicine.

## Graphical abstract

**Figure:**
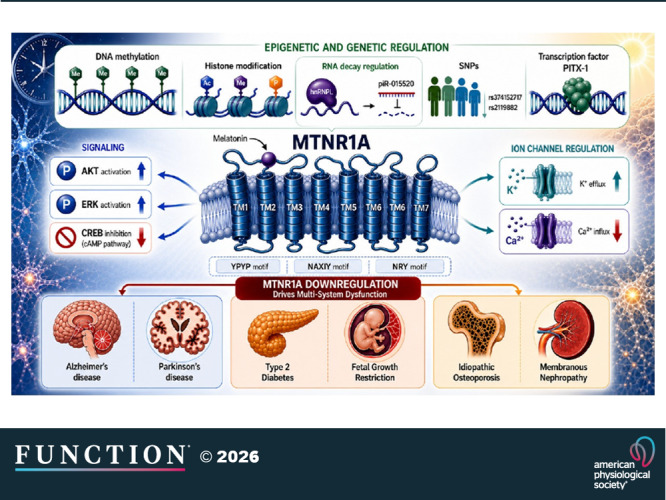

## INTRODUCTION

### Melatonin

Melatonin (N-acetyl-5-methoxytryptamine), originally identified as a pineal gland product, modulates diverse physiological functions. These include the regulation of circadian rhythms, free radical scavenging, anti-inflammatory responses, mitochondrial homeostasis, antioxidant effects, and the enhancement of nitric oxide bioavailability (1). As an indoleamine hormone derived from tryptophan, melatonin is synthesized through a series of enzymatic reactions: tryptophan is first converted to serotonin by tryptophan hydroxylase (TPH) and aromatic l-amino acid decarboxylase (AADC) (2). Subsequently, arylalkylamine-*N*-acetyltransferase (AANAT), the rate-limiting enzyme, converts serotonin to *N*-acetylserotonin (NAS), which is then converted to melatonin by hydroxyindole-O-methyltransferase (HIOMT) (3). The pineal gland serves as the primary source of melatonin, which is secreted into the bloodstream to modulate target cells systemically (4). By signaling through melatonin receptors 1 A (MTNR1A) and 1B (MTNR1B), melatonin regulates a wide array of biological functions via both receptor-dependent and receptor-independent pathways (5).

Melatonin is also produced in various extrapineal tissues, including the gastrointestinal tract, retina, skin, immune cells, bone marrow, and kidneys (6). The expression of key synthetic enzymes, AANAT and ASMT, enables local melatonin production through autocrine and paracrine mechanisms (7). Notably, the gastrointestinal tract represents the largest extrapineal source, containing melatonin levels several 100-fold higher than those in the pineal gland and operating independently of photoperiodic control (8). Moreover, emerging evidence indicates that melatonin is synthesized within mitochondria, where it functions as a potent reactive oxygen species (ROS) scavenger and regulator of mitochondrial homeostasis (9). Together, this ubiquitous distribution highlights melatonin’s dual role as both a circulating hormone and a localized tissue-protective agent, essential for regulating homeostasis across various physiological and pathological contexts.

### MTNR1A Gene Structure

Melatonin receptors are specialized proteins that bind to the hormone melatonin, and in mammals, there are two primary subtypes: melatonin receptor 1 A (MTNR1A) and melatonin receptor 1B (MTNR1B) (10). *MTNR1A* sequence shows less variation than *MTNR1B* among different species, suggesting that *MTNR1A* plays a more fundamental and ancient role (11). The MTNR1A receptor, also known as MT1 or Mel1a, is a G protein-coupled receptor (GPCR) with a characteristic seven-transmembrane (TM) domain structure (2). It is encoded by a gene on human chromosome 4q35.1/4q35.2 that contains two exons, which translate into a 350-amino acid protein (12). This structural similarity leads to a 60% sequence homology with the *MTNR1B* receptor (13). Both *MTNR1A* and *MTNR1B* comprise two exons, yet they differ significantly in genomic span because *MTNR1A* possesses a larger 20 kb intronic region compared with the 11 kb found in *MTNR1B*. This expanded intronic space within the *MTNR1A* gene may harbor a greater density of regulatory elements, enabling more complex and diverse gene expression regulation (14). The conserved two-exon structure of the *MTNR1A* gene shows 90% homology across species such as humans, mice, and rats, reflecting strong evolutionary pressure to preserve its core function (15). These features collectively suggest that *MTNR1A* is more evolutionarily conserved than *MTNR1B*, which could imply a more stable or essential biological role.

### MTNR1A Conserved Sequence Features

**Figure 1.**
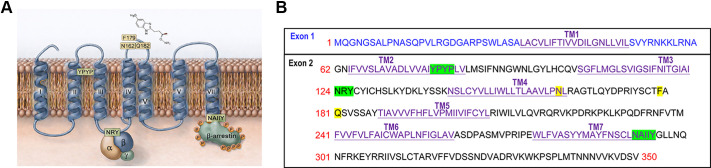
Domain architecture and exon organization of human melatonin receptor 1 A (MTNR1A). *A*: the MTNR1A receptor exhibits a 7-transmembrane (TM) architecture embedded within the plasma membrane. Indicated are critical ligand-binding residues (N162, Q181, and F179) alongside conserved motifs such as YPYP, NRY, and NAXIY. Activation triggers MTNR1A to couple with heterotrimeric G proteins and recruit β-arrestin after phosphorylation, initiating various signaling pathways. *B*: the full-length amino acid sequence highlights the coding regions derived from exon 1 (blue) and exon 2 (black), with residues numbered in red. The 7 TM helices (TM1–TM7) are underlined and color-coded in purple. Key conserved GPCR motifs are emphasized in green, specifically the NRY motif (G-protein activation switch), the YPYP motif (structural folding stabilization), and the NAIIY motif (regulation of active-state stabilization and β-arrestin–mediated internalization). In addition, residues N162, F179, and Q181, which are critical for melatonin binding, are highlighted in yellow. The conceptual framework of A was designed by the author, and the illustration was generated using Gemini.

MTNR1A, like most GPCRs, contains seven transmembrane (TM) α-helices that form a binding pocket for small-molecule ligands such as melatonin (Fig. 1) (15). The N-terminus and several extracellular loops of MTNR1A are responsible for ligand recognition and proper receptor folding. Its C-terminus is shorter, and its intracellular loops more easily interact with G proteins (Gαi/o), Gαq, or β-arrestins to mediate downstream signal transduction than the MTNR1A C-terminus, which can couple to multiple signal transduction cascades (13). The MTNR1A receptor exhibits distinct structural features compared with most GPCRs, including a YPYP motif in its second TM domain, an NRY motif in its third TM, and an NAIIY motif in its seventh TM (Fig. 1) (16). The YPYP motif is essential for maintaining proper MTNR1A folding and stabilizing the transmembrane architecture required for functional receptor expression (Fig. 1) (13). The NRY motif acts as the core activation switch, controlling G-protein coupling and the transition from inactive to active states (Fig. 1) (16). Furthermore, the NAIIY motif regulates the receptor’s active state stabilization and β-arrestin-dependent internalization, thereby shaping downstream signaling bias (Fig. 1) (17). Notably, MTNR1A adopts the NRY motif and NAIIY motif instead of the conventional DRY and NPXXY sequence found in most GPCRs, respectively (17). However, the detailed mechanisms governing MTNR1A’s phosphorylation-dependent internalization, ubiquitin-mediated degradation, and regulatory dimerization remain poorly characterized.

MTNR1A has binding sites for melatonin located at positions between 162 and 181 of the amino acid sequences, with a melatonin affinity EC_50_ of ∼0.5 nM (18). N162 and Q181 form hydrogen bonds with melatonin, whereas F179 contributes to hydrophobic stabilization of the indole ring (Fig. 1) (19). Moreover, the ligand-binding pocket of MTNR1A is narrower than that of typical aminergic GPCRs, which confers high selectivity for melatonin (10). The ligand-dependent endocytosis of MTNR1A from the plasma membrane directs the receptor through the Rab5-positive early endosomes, Rab7-positive late endosomes, and Rab11-positive recycling endosomes, which coordinate receptor sorting, recycling, or lysosomal degradation (20).

## GENE REGULATION OF MTNR1A

### The Transcriptional and Epigenetic Regulation of MTNR1A

**Figure 2.**
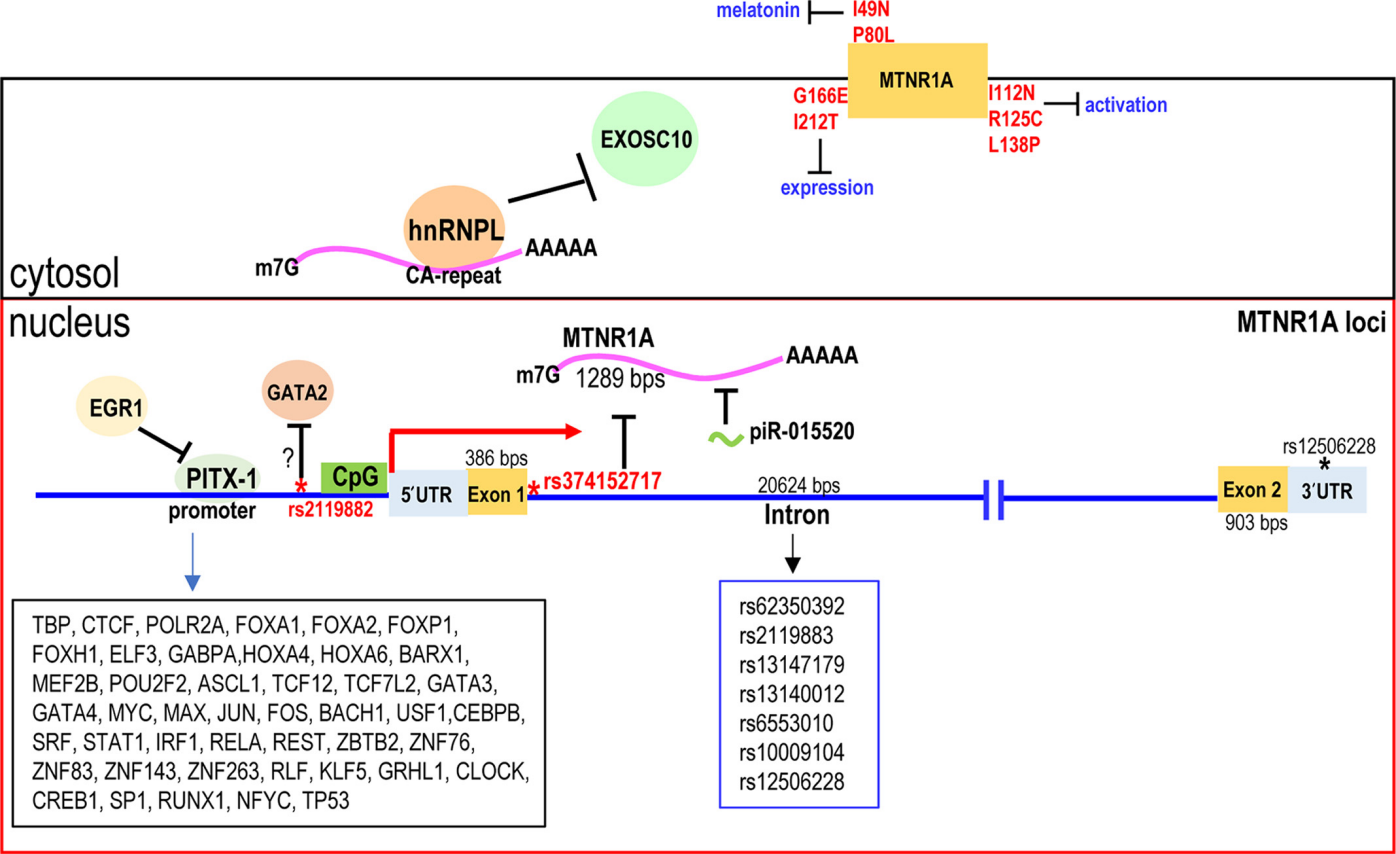
Multilayered regulatory landscape of melatonin receptor 1 A (MTNR1A) from gene expression to protein function. The schematic depicts the MTNR1A genomic architecture, including the lengths of the promoter, 5′UTR, exon 1, intron, exon 2, and 3′UTR within the nucleus. Key cis-regulatory elements in the promoter are highlighted, such as the E-box, PITX-1-binding site, and putative GATA2 motifs adjacent to the CpG island. Clinically and functionally significant single-nucleotide polymorphisms (SNPs) are labeled: coding/promoter variants in red (rs2119882 and rs374152717) and intronic/3′UTR variants in blue square. Black square indicates unvalidated transcription factors within the nucleus, such as TBP, CTCF, POLR2A, the FOXA family, GATA factors, CLOCK, CREB1, SP1, and TP53. The transcript also shows the predicted interaction site for the small RNA piR-015520. The *top* panel details posttranscriptional regulation in the cytosol, featuring hnRNPL binding to CA-repeat sequences and EXOSC10-mediated RNA degradation. Functional profiling of MTNR1A variants reveals distinct molecular defects. The G166E and I212T mutants show severely impaired cell surface expression, whereas I49N exhibits a dual loss of binding activity and surface localization. In contrast, R125C, I112N, and L138P maintain wild-type binding affinities but display reduced G protein and β-arrestin signaling efficacy due to conformational instability. Collectively, this figure illustrates the spatiotemporal control of MTNR1A across the genomic, transcriptomic, and proteomic levels.

The Signaling Pathways Project identified numerous transcription factors [CLOCK, cyclic adenosine monophosphate (cAMP) responsive element-binding protein (CREB), GATA2, and others listed] as being recruited to the *MTNR1A* promoter (Fig. 2) (21, 22). However, the regulatory effect of these factors on promoter activity has not yet been investigated, they have only been confirmed as targets on the *MTNR1A* promoter region. Only pituitary homeobox-1 (PITX-1) has been proven to regulate *MTNR1A* transcriptional activity. Experimental evidence confirms that PITX-1 enhances *MTNR1A* transcriptional activity by directly binding to its promoter in both ovine pars tuberalis cells and mouse gonadotroph cells (23, 24). A chromatin immunoprecipitation experiment further demonstrated that PITX-1 occupies a region of the human *MTNR1A* promoter spanning from −545 to −426 in human renal tubular epithelial cell (TEC) (24). Furthermore, early growth response protein 1 (EGR1) strongly inhibits the *MTNR1A* promoter activity that is stimulated by PITX-1 in a dose-dependent manner (25). This suppression is likely achieved through EGR1 exerting an antagonism of *PITX-1* and chromatin remodeling, rather than simple transcription factor occupancy (23). Together, these results confirm that PITX-1 upregulates *MTNR1A* RNA expression across species.

PITX-1 is a homeobox transcription factor with a well-established role in hindlimb specification and skeletal patterning during embryonic development. Deletion of the mouse Pitx1 gene has been shown to hamper hindlimb cartilage and bone development (26). Functional evidence from human cell models demonstrates that the suppression of PITX-1 induces oncogenic signaling by activating the RAS pathway, whereas its restoration inhibits tumorigenicity in a wild-type RAS-dependent manner (27). Mechanistically, PITX-1 acts as a transcriptional activator of RASAL1, a RAS GTPase-activating protein, thereby downregulating RAS signaling and reducing downstream extracellular signal-regulated kinase (ERK) activation (27). Furthermore, epigenetic silencing of PITX-1 via promoter hypermethylation has been observed in multiple tumor types, suggesting a conserved mechanism of functional inactivation (28). Although PITX-1 increases the transcriptional activity of MTNR1A in TECs, its functional significance in renal pathophysiology has yet to be elucidated.

The presence of a dense CpG island suggests that the *MTNR1A* gene is theoretically prone to hypermethylation and robust circadian regulation (29). Methylation of CpG sites in the *MTNR1A* promoter region is suggested to affect the binding of transcription factors, leading to transcriptional repression (Fig. 2). MTNR1A expression negatively correlated with DNA methylation levels, suggesting that the photoperiod may induce DNA methylation of the *MTNR1A* gene and thus decrease its expression (30). The strong link between photoperiod (day length) and *MTNR1A* DNA methylation demonstrates how environmental factors can induce epigenetic changes. Consistent with this notion, 5-aza-2′-deoxycytidine (a DNA methylation inhibitor) reactivated *MTNR1A* expression in oral cancer cells where the gene had been silenced via DNA methylation (31).

Although MTNR1A expression in the mouse suprachiasmatic nucleus (SCN) exhibits a low-amplitude circadian rhythm, its promoter does not contain functional canonical E-box motifs (5′-CACGTG-3′) (32). In contrast to MTNR1B, which is a direct transcriptional target of the core circadian clock, MTNR1A is not directly regulated by the BMAL1/CLOCK heterodimer (25, 33, 34). Instead, the rhythmic expression of MTNR1A is likely mediated through indirect mechanisms, such as secondary clock loops involving ROR/REV-ERB response elements or tissue-specific transcriptional regulators (25, 33). These results suggest that the molecular mechanism driving the MTNR1A rhythm is an indirect pathway or downstream effect, rather than a classic direct binding interaction of core clock genes to the promoter.

Hallmarks of active enhancers and promoters, including histone H3 lysine-4 monomethylation (H3K4me1), H3K4me3, and H3K27ac, were found to be enriched around the *MTNR1A* promoter based on data from the UCSC Genome Browser (35). These histone marks suggest that in tissues where MTNR1A is expressed via its promoter, the chromatin is in an active state. Moreover, the histone deacetylase (HDAC) inhibitor valproic acid was shown to increase MTNR1A levels in C6 glioma cells, accompanied by decreased mRNA levels of methyl-CpG-binding protein 2 (MeCP2) and HDACs (36). These results suggest that MeCP2 and HDACs may repress MTNR1A by binding methylated DNA and deacetylating histones. Together, DNA methylation and chromatin marks are key modulators of MTNR1A expression.

### Posttranscriptional Regulation of MTNR1A

A very limited number of factors have been shown to regulate MTNR1A expression at the RNA level. A previous study indicated that the cytoplasmic heterogeneous nuclear ribonucleoprotein L (hnRNPL) protein stabilized the MTNR1A transcript through CA-repeat elements located in its coding region in TECs (37). The interaction between hnRNPL and MTNR1A serves to protect MTNR1A RNA degradation by the exosome component 10 (EXOSC10) protein (Fig. 2). The increase in MTNR1A levels observed at midnight correlated with robust binding between cytoplasmic hnRNPL and the MTNR1A RNA (37). Posttranscriptional regulation of the MTNR1A transcript can occur via noncoding RNAs. However, no single microRNA (miRNA) has been identified as the key regulator of MTNR1A. Notably, a PIWI-interacting RNA (piRNA), piR-015520, was found to decrease MTNR1A expression by binding to a genomic region in HEK293 cells (38). Bioinformatic analyses have predicted several miRNA-binding sites in the 3′-UTR (untranslated region) of *MTNR1A*, including miR-19 or let-7, but direct miRNA repressors of MTNR1A are still being investigated.

After MTNR1A is expressed, its protein stability can be modulated by posttranslational modifications (PTMs). MTNR1A undergoes ligand-induced ubiquitination and is targeted to the endo-lysosomal degradation pathway following melatonin stimulation in lung adenocarcinoma cells (39). The ubiquitin-specific protease 8 (USP8) removes ubiquitin from MTNR1A, thereby preventing its lysosomal degradation and stabilizing the receptor (39). However, the detailed mechanism of the ubiquitination site(s) in MTNR1A upon melatonin binding has not been identified. Moreover, other PTMs of MTNR1A, including phosphorylation, glycosylation, or palmitoylation, have been less documented. No other deubiquitinase (DUBs), E3 ligases, or trafficking proteins have been experimentally confirmed to regulate MTNR1A degradation.

### MTNR1A Expression Under Stress Inflammation

Although it is established that melatonin inhibits NF-κB signaling via MTNR1A, it remains to be elucidated whether proinflammatory factors modulate the expression of the MTNR1A receptor (40). Estradiol also downregulates MTNR1A expression in the ovarian membrane through an ER-dependent repression mechanism in vivo (41). Bisphenol A is reported to decrease MTNR1A levels in Leydig and intestinal cells (42). Similarly, alcohol significantly decreases the expression of MTNR1A in the outer nuclear layer of rat retina tissue (43). Lipopolysaccharide (LPS) injection reduce MTNR1A protein expression in activated microglia in the corpus callosum after 6 h of treatment; this downregulation can be reversed by subsequent melatonin treatment (44, 45). Notably, treatment with NG-497 (an adipose triglyceride lipase inhibitor) was found to increase MTNR1A protein levels without substantially altering its mRNA expression, suggesting a posttranscriptional mechanism (46). Moreover, the NG-497’s anti-inflammatory and neuroprotective properties were found to be dependent on MTNR1A levels (46). These results suggest that NG-497 functions as an MTNR1A-targeting compound to deliver its beneficial effects.

### MTNR1A-Mediated Signaling Pathways

MTNR1A is known to form homodimers with itself and heterodimers with other GPCRs, which results in altered signaling properties in a context-specific manner (47). The formation of MTNR1A/MTNR1A homodimers is indicated 3 or 4 times more frequent than the formation of MTNR1A/MTNR1B heterodimers or MTNR1B/MTNR1B homodimers, indicating a strong preference for MTNR1A homodimerization (48). GPR50, an orphan GPCR structurally related to melatonin receptors, has also been shown to heterodimerize with MTNR1A (49). This interaction inhibits melatonin binding and subsequent signaling, indicating that GPR50 functions as a negative regulator of MTNR1A activity (50). Together, this MTNR1A homodimerization is critical for initiating C-terminus-mediated signaling.

**Figure 3.**
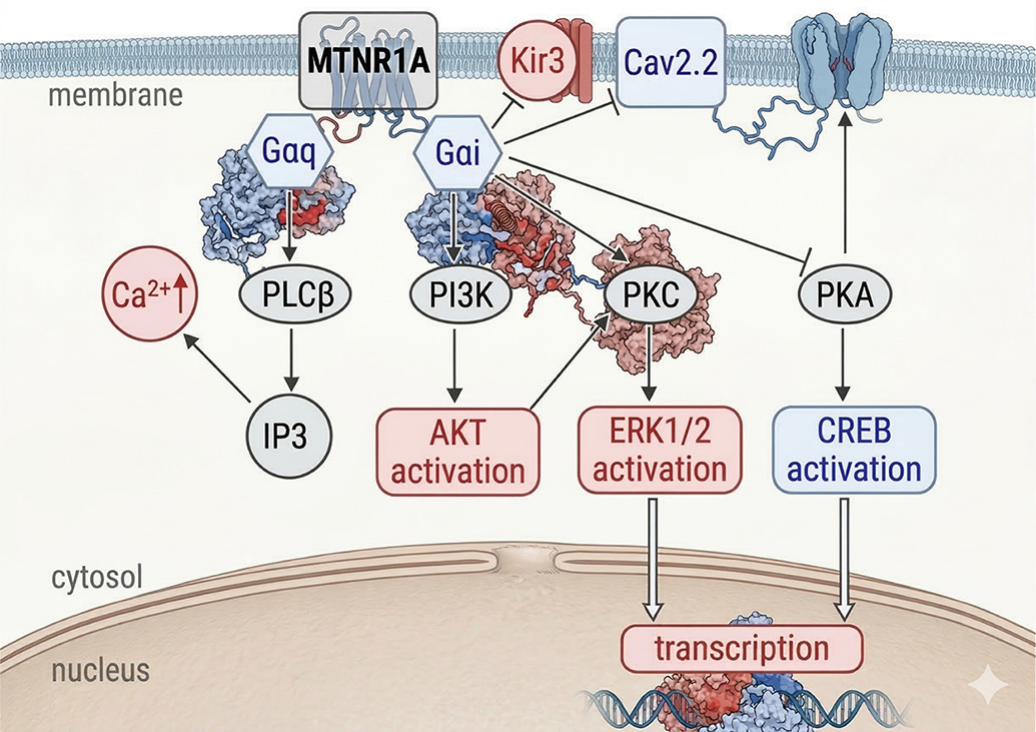
Schematic overview of melatonin receptor 1 A (MTNR1A)-mediated signaling pathways and downstream cellular effects. MTNR1A activates multiple G-protein-dependent signaling cascades. Via Gαi, MTNR1A suppresses PKA activity while simultaneously activating PI3K/AKT and PKC/ERK1/2 signaling, which results in transcriptional regulation. Through Gαq, MTNR1A stimulates PLCβ, leading to IP3 production and increased intracellular Ca^2+^ levels. MTNR1A also modulates several ion channels, including Kir3 (inward rectifier K^+^ channels), Cav2.2 (voltage-gated Ca^2+^ channels), and KCa1.1 (large-conductance Ca^2+^-activated K^+^ channels). The conceptual framework of this figure was designed by the author and the illustration was generated using Gemini.

The intracellular C-terminus of MTNR1A, together with its third intracellular loop, interacts with distinct signaling partners, including Gαi, Gαq, and β-arrestins, thereby mediating multiple downstream pathways such as the cAMP/PKA, phospholipase C (PLC)/PKC, and MAPK/ERK cascades (16). MTNR1A signals primarily through Gαi to inhibit the cAMP/PKA pathway and suppress CREB-dependent gene expression, as observed in rat hippocampal progenitor cells and pituitary models (Fig. 3) (51). Moreover, in HEK293 cells expressing human MTNR1A, the receptor stimulates Gαi-dependent phosphatidylinositol 3′-kinase (PI3K)/protein kinase B (AKT) and protein kinase C (PKC)/ERK1/2 signaling (Fig. 3) (52). MTNR1A also couples to Gαq to activate the phospholipase Cβ (PLCβ)/inositol 1,4,5-triphosphate (IP3) pathway, which leads to the upregulation of intracellular Ca^2+^ in osteoblasts (Fig. 3) (53). Moreover, MTNR1A receptor mediates the activation of K^+^ channels (Kir3) and Ca^2+^ channels (Cav2.2) in neuronal tissues (Fig. 3) (54). In general, MTNR1A mediates intracellular signaling mainly via the Gαi pathway rather than the Gαq pathway (55). However, BRET and functional assays have demonstrated that MTNR1A can switch between inhibitory Gαi signaling and activating Gαq responses depending on its receptor dimeric state (56). Although MTNR1A couples to both Gαi and Gαq pathways, MTNR1B signals primarily through Gαi and exhibits little or no Gαq-mediated Ca^2+^/PKC activation.

The association between MTNR1A and β-arrestin significantly enhances the phosphorylation of PKA at Thr197 and ERK at Thr202 and Tyr204 in transfected HEK293T cells (57). MTNR1A reduces CREB-target genes involved in circadian rhythms, metabolism, and hormone secretion via the downregulation of cAMP levels and CREB phosphorylation in the Gαi/PKA pathway within the suprachiasmatic nucleus (SCN) and pars tuberalis (58). Through the Gαq/PLCβ pathway, MTNR1A increases the activity of mitochondrial biogenesis and antioxidant defenses in brown adipose tissue (59). In the ERK/MAPK pathway, MTNR1A mediates cell survival and growth responses through immediate early genes such as FOS and EGR1 in hippocampal and neural lineage cells (10). Although signaling outputs through CREB and ERK are well established, the direct transcriptional targets regulated downstream of MTNR1A remain largely undefined. Identifying these genes will require MTNR1A-dependent transcriptomics combined with CREB/ERK motif analysis and chromatin immunoprecipitation-based validation.

### MTNR1A Tissue Distribution

**Figure 4.**
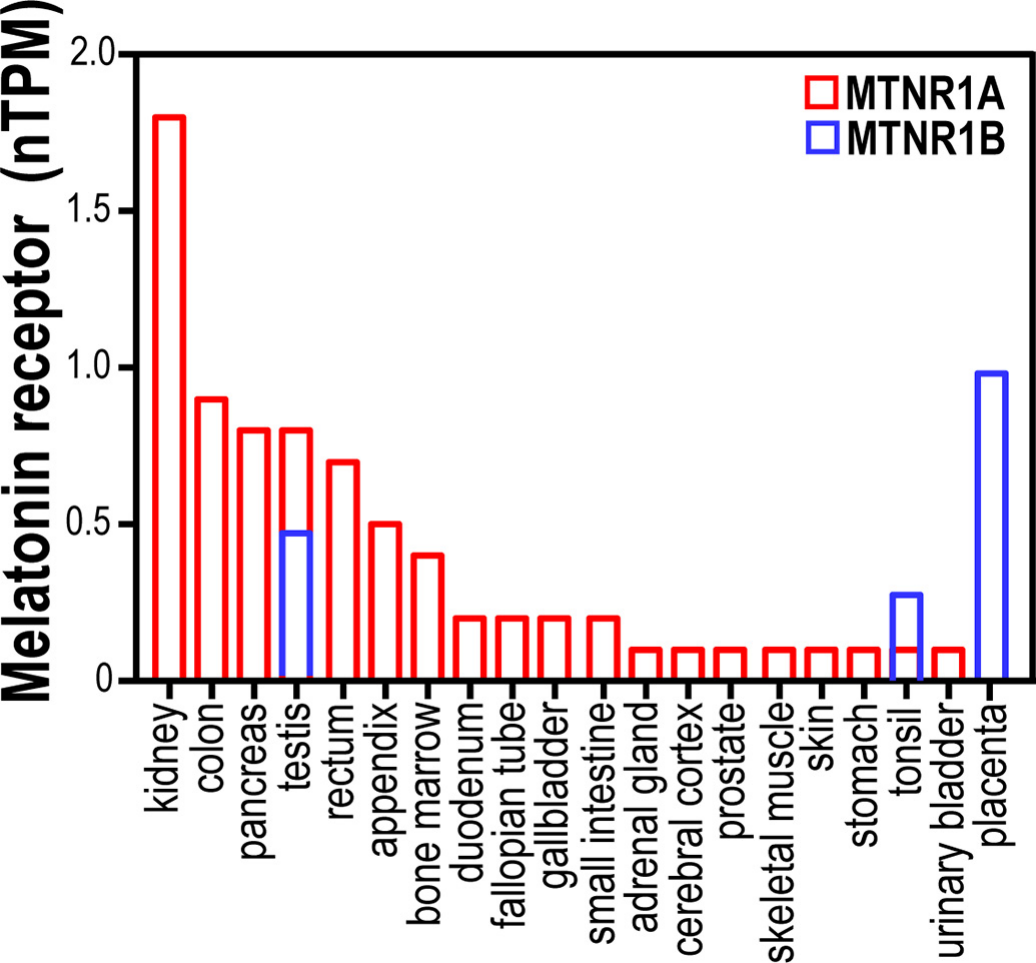
Tissue-specific expression profile of melatonin receptor 1 A (MTNR1A) and MTNR1B in human organs. A bar plot showing the normalized transcripts per million (nTPM) of MTNR1A (red) and MTNR1B (blue) across multiple human tissues. Consensus transcript expression levels, based on transcriptomics data from The Human Protein Atlas version 24.1 and Ensembl version 109, are summarized for the 20 tissues where MTNR1A expression was detectable.

Both MTNR1A and MTNR1B are widely expressed in peripheral tissues, but most tissues predominantly express either MTNR1A or MTNR1B (60, 61). According to the Human Protein Expression Atlas Database, MTNR1A and MTNR1B RNA are detectable in 19 and 4 of the 40 surveyed tissues, respectively (Fig. 4) (62, 63). Notably, the kidney exhibits the highest MTNR1A expression among these 20 tissues, which also include the colon, pancreas, testis, cerebellum, rectum, appendix, bone marrow, duodenum, fallopian tube, gallbladder, small intestine, adrenal gland, cerebral cortex, prostate, skeletal muscle, skin, stomach, tonsil, and urinary bladder (Fig. 4). Conversely, MTNR1B shows the highest expression found in the placenta, followed by the testes, and then the tonsils (Fig. 4). Interestingly, the placenta express high levels of MTNR1B but no MTNR1A, whereas the kidney expresses high levels of MTNR1A but no MTNR1B (Fig. 4). The divergent tissue distribution of MTNR1A and MTNR1B allows melatonin signaling to precisely modulate cellular physiology in a tissue-specific manner.

In the kidneys, MTNR1A RNA and protein are predominantly expressed in renal tubular epithelial cells (TEC), but to a lesser extent in glomerular cells (24). But, MTNR1B RNA expression was undetectable in TEC and glomerulus (24). A similar notion exists in the pancreas, where higher levels of MTNR1A mRNA are observed in alpha cells compared with MTNR1B mRNA (64). MTNR1A expression in the testis is limited to strong perinuclear staining in primary spermatocytes and weak staining in Leydig cells (65). Conversely, MTNR1B is broadly expressed across all spermatogenic and supporting cell populations (65). MTNR1A expression in brain tissue is localized to the suprachiasmatic nuclei of the hypothalamus and other regions, including the retina, hippocampus, amygdala, and substantia nigra (54). MTNR1A is expressed throughout the gastrointestinal tract, mainly in epithelial and enteroendocrine cells of the stomach and intestine, but its levels vary by segment and are generally lower than MTNR1B (66). The MTNR1A receptor is expressed in the thymus and the spleen, as well as in CD4, CD8, and B cells, whereas MTNR1B receptors were not detected in any of these cells (67).

MTNR1A shows relatively higher mRNA expression in certain tissues such as the kidney, colon, and testis (Fig. 4). Conversely, its expression is very low or undetectable in many other tissues, including the stomach, endocrine glands, muscle, and skin (Fig. 4). Protein-level data are currently limited and require further confirmation from immunohistochemical images. The widespread yet specific tissue distribution of MTNR1A indicates that the pleiotropic effects of melatonin are not solely attributable to systemic melatonin levels but are also linked to local receptor expression, which enables tissue-specific responses. Understanding the tissue and cellular distribution of MTNR1A is crucial for elucidating the full spectrum of melatonin’s physiological roles and its significance in various pathological conditions.

### MTNR1A Knockout Mice

Previous studies have observed multiple phenotypes in the *Mtnr1a* global knockout (KO) mice, including systemic metabolic dysfunction, central circadian rhythm defects, neuropsychiatric behavioral defects, and ocular structural degeneration. To date, two knockout mouse strains are available: the B6.129S4-*Mtnr1a*^tm1Rep/J^ strain from The Jackson Laboratory, which was generated by disrupting *Mtnr1a* exon 1 in embryonic stem (ES) cells using a PGK-neomycin cassette before injecting correctly targeted ES cells into C57BL/6 blastocysts, and the C57BL/6Smoc-*Mtnr1A*^em1Smoc^ strain from the Shanghai Model Organisms Center, Inc., which was created using CRISPR/Cas9 to delete *Mtnr1a* exon 2. However, floxed mice are not available to generate tissue-specific knockouts.

Genetic deletion of *Mtnr1a* results in behavioral abnormalities, including depression-like phenotypes and deficits in sensorimotor gating (68). In addition, *Mtnr1a* knockout mice exhibit impaired melatonin-mediated regulation of suprachiasmatic nucleus (SCN) neuronal activity and circadian phase shifting (69). Although the Mtnr1a receptor mediates high-affinity melatonin binding and acute SCN inhibition, Mtnr1b exerts distinct and mechanistically separable pathways that modulate SCN circadian physiology (70). Impaired PI3K activity and insulin resistance were observed in the liver, skeletal muscle, and adipose tissue of *Mtnr1a* knockout mice (71). Finally, *Mtnr1a* knockout mice exhibited loss of photoreceptor nuclei in the outer nuclear layer and a decreased number of ganglion cells compared with control mice at 18 mo (72). Although *Mtnr1a* global deletion mice exhibit certain physiological defects, these systemic phenotypes may not be solely attributable to the absence of *Mtnr1a* in a single tissue. Therefore, the use of tissue-specific knockout mice is necessary to delineate the specific contributions of MTNR1A in different organs, especially those with higher MTNR1A levels.

Despite a high protein sequence identity of ∼80% between human MTNR1A and mouse Mtnr1a (73), their promoter sequences exhibit low similarity, suggesting distinct regulatory mechanisms. Furthermore, melatonin signaling via these receptors typically facilitates nighttime activity in rodents while promoting rest in humans (74). Unlike the relatively CNS-restricted expression profile observed in mice (75), human MTNR1A is extensively distributed across peripheral tissues, including the renal, pancreatic, and colon (Fig. 4). Mouse strains vary significantly in their melatonin phenotypes, ranging from melatonin-deficient lines, such as C57BL/6, to melatonin-proficient ones, such as C3H/HeN. These inherent differences can complicate the phenotypic interpretation of knockout (KO) models (76). Although *Mtnr1a* KO mice are vital for mechanistic studies, species-specific signaling and tissue distribution differences must be considered when translating these results to human metabolic and peripheral pathologies.

## MTNR1A POLYMORPHISMS AND DISEASE PATHOGENESIS

### MTNR1A Single-Nucleotide Polymorphism

Single-nucleotide polymorphisms (SNPs) within MTNR1A modulate receptor activity, thereby influencing a range of physiological traits and disease susceptibilities. Representative SNPs correlate with specific pathophysiological pathways of melatonin signaling, as detailed in Fig. 2. The association of rs12506228 with clinical Alzheimer-type dementia was found in a very elderly population (77). In vitro knockdown of MTNR1A increased the amyloidogenic processing of amyloid precursor protein (APP) (77). Furthermore, the correlation between rs12506228 and slower socioemotional and communication development during the first 8 mo of infancy may be linked to the importance of MTNR1A during fetal development (78). Three MTNR1A SNPs (rs62350392, rs2119883, and rs13147179) showed linkage and association to type 2 diabetes in Italian families (79). The MTNR1A polymorphism rs2119882 has been widely investigated. Studies indicate that it is associated with an increased risk of polycystic ovary syndrome and gestational diabetes mellitus in Han Chinese women, correlating with higher fasting plasma glucose levels (79, 80). Although SNP variants in MTNR1A may be linked to type 2 diabetes risk, the evidence is weaker compared with MTNR1B (80). MTNR1A rs2119882 was more prone to developing distant metastasis in Hepatocellular carcinoma (81). Further analyses identified that rs2119882 is located on the consensus binding site of the GATA2 transcription factor within the promoter region of the MTNR1A gene (Fig. 2). There is also a correlation between the expression levels of GATA2 and MTNR1A using TCGA datasets (81). It remains to be proven whether rs2119882 abolishes GATA2 recruitment and consequently decreases MTNR1A expression.

MTNR1A rs13140012 and rs6553010 might contribute to the ability to predict advanced stage in oral cancer and urothelial cell carcinoma, respectively (82, 83). Moreover, SNP rs13140012 in intron 1 of MTNR1A was significantly associated with calcium nephrolithiasis (84). But, no significant association was found between serum melatonin and rs13140012 in patients with end-stage renal disease (85). The effect of a paternally inherited genetic variant (rs10009104) on the comorbidity of asthma and allergic rhinitis is mediated by differential DNA methylation at the CpG site (cg02303933) located within the MTNR1A gene (86). Notably, most studies identified the correlation between disease status and MTNR1A SNPs, but none have demonstrated experiments to prove it. However, the MTNR1A variant rs374152717 as a genetic cause of idiopathic osteoporosis (IOP) in Ashkenazi Jewish young individuals has been demonstrated in a mouse model (87). This MTNR1A rs374152717 variant, located in a donor-splicing site, led to downregulation of functional MTNR1A protein and subsequent dysregulation of melatonin signaling by inducing the low bone mass phenotype (87).

A key genetic observation in these studies is that many clinically impactful MTNR1A SNPs are predominantly located in introns or noncoding regulatory regions rather than within the coding sequence. This distribution suggests that MTNR1A variation does not arise from large structural alterations or changes in the ligand-binding properties of the MTNR1A receptor itself, but instead from subtle defects in the spatiotemporal regulation of MTNR1A levels. Therefore, MTNR1A expression levels are critically important, and most variants exert their pathogenic effects by modifying transcription factor binding, epigenetic regulation, or RNA splicing, thus altering the MTNR1A protein expression and subsequent melatonin signaling. Since MTNR1A is expressed in a wide range of tissues, SNPs in this gene should simultaneously influence multiple seemingly unrelated disease categories, including neurodegenerative diseases, metabolic and endocrine dysregulation, tumorigenesis, and idiopathic osteoporosis. Future studies could use CRISPR-Cas9 technology to introduce these clinically associated SNPs into cell models to further characterize and validate their functional effects.

### MTNR1A Variants and Mutants

A previous study identified six nonsynonymous MTNR1A mutations in blood samples from patients with autism spectrum disorders (ASDs) (88). Of particular interest are the I49N mutant, which loses melatonin-binding activity and cell surface expression, and the MTNR1A G166E and I212T mutants, which showed severely impaired cell surface expression (88). However, the clinical manifestations associated with these mutants remain mild, likely due to compensation from other MTNR1A receptors or neuronal network plasticity. Furthermore, the overall frequency of these mutants is comparable with that of control individuals (50). This suggests that although these variants are functionally deleterious, their mild clinical impact means they do not represent a major risk factor for ASD.

Thirty-six nonsynonymous variants, including 34 rare ones, were identified in MTNR1A from a cohort of 8,687 blood DNA samples across several population studies (19). One variant, P80L, is located within the conserved YPYP motif and results in a complete loss of melatonin binding, representing a full loss-of-function mutation. Three other variants (R125C, I112N, and L138P) can still bind melatonin with dissociation constant values comparable with the wild type. However, their active conformations are unstable, leading to reduced efficacy in both G protein activation and β-arrestin recruitment. Specifically, R125C disrupts the critical NRY motif, resulting in a total loss of signaling. I112N and L138P interfere with the PIF motif (TM3-TM5-TM6 network), thereby destabilizing the active receptor conformation. Although these variants show no significant association with diabetes or obesity, they may cause pronounced signaling defects in cells that exclusively express MTNR1A, a possibility that warrants further investigation (19).

Why do these MTNR1A variants completely lose melatonin binding yet show no evident clinical abnormalities? This suggests that melatonin signaling can be compensated by MTNR1B or other GPCRs, thus maintaining key downstream pathways. Furthermore, MTNR1A may exhibit a low level of constitutive Gα activity even without ligand stimulation. Therefore, a small amount of signaling can persist even when melatonin binding is lost. Moreover, since these variants are extremely rare (minor allele frequency < 0.00005), there are currently insufficient samples to fully assess their clinical impact. Further study should focus on MTNR1A-solely expressing tissue to dissect the role of specific variants or mutants in disease status.

## DYSREGULATION OF MTNR1A IN DISEASES

### Downregulation of MTNR1A in Idiopathic Osteoporosis

The MTNR1A expression is significantly decreased in several pathological conditions, suggesting that MTNR1A-mediated endogenous protection is compromised in these states (Table 1). A recent study indicated that the rs374152717 splice-site mutation disrupts normal MTNR1A pre-mRNA splicing, generating unstable or truncated transcripts that undergo rapid degradation, thereby triggering decreased MTNR1A expression in idiopathic osteoporosis (87). A knock-in mouse model carrying the human splice-site mutation reproduced the early-onset low bone mass phenotype, which was caused by impaired osteoblast differentiation, reduced bone formation, and increased osteoblast senescence (87). In these MTNR1A rs374152717 splice-site mutation knock-in mice, MTNR1A could no longer suppress adenylate cyclase, causing abnormally elevated cAMP levels and overactivation of the PKA pathway (87). This is a well-studied SNP example so far, in which rs374152717 reduces MTNR1A protein and removes melatonin’s brake on the cAMP/PKA pathway then driving osteoblast dysfunction and osteoporosis.

**Table 1.** Summary of diseases associated with altered MTNR1A expression

Disease	Affected Cells	Pathological Consequence	Key Molecular Mechanism
Idiopathic osteoporosis	Decreased in osteoblasts	Low bone mass; compromised bone turnover; senescence of osteoblasts; impaired differentiation	Induction of adenylate cyclase, causing abnormally elevated cAMP levels and overactivation of the PKA pathway
Membranous nephropathy	Decreased in TECs	Impaired TEC survival and increased risk of membranous nephropathy progression	Induction of PKA/CREB pathway associated with decreased CDH1 expression and increased levels of Per2 and α-SMA expression
Ductal breast cancer	Decreased in tumor cells	Prognostic factor for overall survival and event-free survival in estrogen receptor-positive tumors	Unknown
Colorectal cancer	Decreased in tumor cells	Correlated with cryptochrome 1 expression	Unknown
Renal carcinoma	Decreased in tumor cells	Negative association with MMP-9 expression; correlates with markedly worsened overall survival	Unknown
Oral squamous cell carcinoma	Decreased in tumor cells	Correlated with tumor size and shorter overall survival	Unknown
Gliomas and glioblastoma	Decreased in tumor cells	The MTNR1A/MTNR1B expression ratio is a positive prognostic factor in gliomas	Unknown
Gastric adenocarcinoma	Increased in tumor cells	Associated with lymph-node metastasis and significantly poorer overall survival	Unknown
Fetal growth restriction	Decreased in the placenta	Impaired placental function; fetal developmental delay	Unknown
Type 2 diabetes	Decreased in hepatocytes	Accumulation of fat mass and systemic insulin resistance	Activation of phosphatidylinositol-3-kinase
Parkinson’s disease	Decreased in both the substantia nigra and the amygdala	Accumulation of α-synuclein due to impaired LC3-associated phagocytosis	Downregulation of ferroptosis resistance
Alzheimer’s disease	Decreased in the suprachiasmatic nucleus	Accumulation of amyloid-beta plaques	Increased the amyloidogenic processing of the amyloid precursor protein

This table compiles reported disorders in which MTNR1A expression is dysregulated across specific cell types. Reduced MTNR1A levels are linked to impaired osteoblast turnover in idiopathic osteoporosis, decreased tubular epithelial cell survival in membranous nephropathy, and poorer prognosis across several cancers, including ductal breast cancer, colorectal cancer, renal carcinoma, oral squamous cell carcinoma, and gliomas. Increased MTNR1A expression is observed in gastric adenocarcinoma, whereas placental MTNR1A reduction contributes to fetal growth restriction. The table summarizes the molecular pathways for some of these diseases, whereas others remain unknown. α-SMA, α-smooth muscle actin; MMP-9, matrix metalloproteinase; MTNR1A, melatonin receptor 1; TEC, tubular epithelial cell; PER2, period 2.

### Downregulation of MTNR1A in Membranous Nephropathy

MTNR1A levels are significantly downregulated in renal tubular epithelial cells (TECs) in both clinical membranous nephropathy (MN) and experimental MN models (24). Since pituitary homeobox-1 (PITX-1) promotes MTNR1A expression via direct binding to its promoter, the observed decreased PITX-1 levels in MN samples likely cause the low MTNR1A levels. Knockdown of MTNR1A, PITX-1, or cyclic adenosine monophosphate-responsive element-binding protein (CREB) all lead to decreased E-cadherin (CDH1) expression and increased levels of period circadian regulator 2 [period 2 (PER2)] and α-smooth muscle actin (αSMA) expression (24). Blockade of the MTNR1A receptor with luzindole in MN mice further impaired renal function associated with CDH1 downregulation and PER2 and αSMA upregulation (24). Low PITX1-driven downregulation of MTNR1A promoted TEC injury in MN, primarily by increasing PKA/cAMP pathway activity.

### Downregulation of MTNR1A in Cancers

The downregulation of MTNR1A expression has been documented in breast cancer, oral squamous cell carcinoma (OSCC), colorectal cancer, and renal cell carcinoma (RCC) (31, 89, 90). In colorectal cancer, a decrease in MTNR1A expression was observed, which also correlated with cryptochrome 1 (Cry1) expression (90). The low MTNR1A level was identified in triple-negative breast cancer, and MTNR1A levels serve as an independent prognostic factor for overall survival and event-free survival in estrogen receptor (ER) tumors (89). Interestingly, MTNR1A receptor overexpression both inhibits MCF-7 cell proliferation and enhances their sensitivity to melatonin’s antiproliferative effect (91). In OSCC cell lines, MTNR1A levels were found to be decreased but were restored after treatment with 5-aza-2′-deoxycytidine (31). In clinical OSCC tumors, MTNR1A levels were significantly negatively associated with tumor size and a shorter overall survival (31). MTNR1A expression is significantly reduced in RCC and negatively associated with MMP-9 expression, exhibiting markedly worse overall survival (92). High MTNR1A levels in gastric adenocarcinoma (GC) are strongly associated with lymph node metastasis and significantly poorer overall survival, suggesting that elevated MTNR1A increases mortality risk (93). Except for gastric adenocarcinoma, results from other cancer types suggest that restoring MTNR1A levels tends to slow tumor progression, but the detailed mechanism of how decreased MTNR1A contributes to cancer should be explored.

### Downregulation of MTNR1A in Type 2 Diabetes

Although the majority of studies have identified an altered MTNR1B as a genetically validated target for type 2 diabetes (T2D) progression, it is MTNR1A that shapes the metabolic phenotype of T2D by modulating hepatic insulin sensitivity (94). Decreased expression of MTNR1A has been reported in the liver of individuals with T2D. Mice lacking *MTNR1A* knockout exhibit impaired skeletal muscle glucose uptake, accumulated fat mass, and marked systemic insulin resistance via the activation of phosphatidylinositol-3-kinase (PI3K) (94). However, further study should use tissue-specific knockout mice to avoid confounding systemic metabolic effects that result from a total *MTNR1A* knockout.

### Downregulation of MTNR1A in Parkinson’s Disease

The downregulation of MTNR1A and MTNR1B expression in both the substantia nigra and the amygdala with Parkinson’s disease (PD) (95). Alpha-Syn preformed fibrils (PFFs) induce iron accumulation and ferroptosis in PD model, which is markedly worsened in *MTNR1A*-knockout mice (96). Loss of MTNR1A suppresses the Sirt1/Nrf2/HO-1/Gpx4 antioxidant-ferroptosis-resistance pathway, lowering neuronal protection. Conversely, overexpression of MTNR1A reversed these effects (96). In line with this notion, the loss of MTNR1A in microglial cells exacerbates the aggregation of α-Syn in neurons induced by PFF α-Syn. Mechanistically, MTNR1A upregulates Rubicon-dependent LC3-associated phagocytosis (LAP) for α-syn clearance (97). Together, MTNR1A emerges as a central neuroprotective receptor in PD by enhancing resistance to ferroptosis and enabling microglia to clear pathological α-syn via LC3-associated phagocytosis.

### Downregulation of MTNR1A in Alzheimer’s Disease

MTNR1A mediates the receptor-dependent effects of melatonin on hippocampal synaptic plasticity, a mechanism that facilitates improvements in spatial learning and memory in an Alzheimer’s disease (AD) mouse model (98). Of note, melatonin’s actions in reducing amyloid plaque burden, lowering Aβ1-42 levels, and preserving mitochondrial complex IV activity are achieved in a MTNR1A receptor-independent manner (98). Thus, MTNR1A is essential for the cognitive and behavioral modulation by melatonin but not for its core biochemical or antiamyloid effects (98). Furthermore, RNA interference (RNAi)-mediated silencing of MTNR1A increased the amyloidogenic processing of amyloid precursor protein (APP) in N2A neurons, whereas its overexpression produced the opposite effect (77). These findings collectively suggest that weaker physiological melatonin signaling contributes to the pathological cascade of AD (77). From a genetic perspective, the hypothesis that the rs12506228 variant acts as a regulatory element modulating MTNR1A promoter methylation and subsequent gene downregulation warrants further experimental validation (78).

### Downregulation of MTNR1A in Fetal Growth Restriction

Both MTNR1A and MTNR1B receptor expression is significantly decreased in placentas from pregnancies complicated by fetal growth restriction (99). Although the decline of MTNR1A is most pronounced in the apical syncytiotrophoblast, which is the surface layer directly exposed to maternal circulation, the detailed mechanism is not clear (99). Gliomas show significantly decreased MTNR1A and increased MTNR1B expression compared with normal cortex, and a high MTNR1A/MTRN1B ratio strongly predicts better patient survival (100). Selective melatonergic drugs that activate MTNR1A and inhibit MTNR1B robustly block tumor growth in vitro and reduce intracranial tumor burden in vivo (100).

## CONCLUSIONS AND FUTURE DIRECTIONS

The MTNR1A receptor is expressed across various tissues, including the cerebellum, testes, rectum, appendix, and bone marrow; however, notably higher expression levels are localized within the kidney, colon, and pancreas (Fig. 4). However, detailed studies deciphering the tissue-specific roles of MTNR1A remain scarce. The regulation of MTNR1A expression has primarily focused on transcriptional regulation, involving factors like PITX-1 and DNA methylation, and posttranscriptional regulation, involving noncoding RNAs such as piR-015520 and proteins like hnRNPL (24). Further investigation is needed to explore the mechanisms of translation and posttranslational regulation of MTNR1A. Specifically, the processes involved in the degradation and recycling of the MTNR1A protein require detailed study. Moreover, the C-terminal-mediated downstream signaling triggered by the activation of MTNR1A with its ligand, melatonin, also needs to be thoroughly explored.

Decreased levels of the MTNR1A receptor have been observed in several diseases, including membranous nephropathy (MN), breast cancer, colorectal cancer, renal carcinoma, oral squamous cell carcinoma, gliomas, and fetal growth restriction (Table 1). However, most studies have only demonstrated the downregulated levels of MTNR1A in these diseases without further dissecting the receptor’s specific role. Only the decrease of MTNR1A in MN has been explored in depth using the MTNR1A antagonist luzindole to block MTNR1A-mediated downstream signaling (101). Luzindole treatment in mice worsened renal function, evidenced by a marked increase in proteinuria and hypercholesterolemia and a significant decrease in serum albumin (24). Notably, the molecular mechanism identified that decreased albumin levels lowered PITX1 expression, which, in turn, triggered the downregulation of MTNR1A in renal tubular epithelial cells (TECs). Since luzindole treatment lacks specificity for analyzing MTNR1A in renal TECs, future studies using kidney-specific *MTNR1A* knockout mice will be critical for extending the current understanding of how the MTNR1A receptor acts against MN progression.

Several clinical diseases are associated with MTNR1A single-nucleotide polymorphisms (SNPs). However, most studies focus only on identifying the correlation between disease severity and the MTNR1A SNP. Only the rs374152717 variant has been studied in knock-in mice, where it successfully reproduced the low bone mass phenotype found in young adult patients with idiopathic osteoporosis (IOP). Moreover, mice carrying the rs374152717 variant exhibited dysregulated melatonin signaling due to atypical changes in the cAMP, calcium, and MAPK signaling pathways, ultimately leading to the induction of senescence in osteoblasts. The rs374152717 variant, which is located at a splicing site, results in a splicing error. This error produces an unstable protein fragment, leading to decreased MTNR1A levels in osteoblasts. These studies provide evidence that the MTNR1A rs374152717 variant functions as a loss-of-function allele that impairs bone turnover by inducing senescence in osteoblasts in IOP.

Ferroptosis is an iron-dependent form of regulated cell death driven by polyunsaturated fatty acid (PUFA) oxidation and GPX4 deficiency, contributing to various clinical conditions (102). Emerging evidence suggests that low MTNR1A expression and the induction of ferroptosis occur across multiple diseases (103). These include normal cell death in cancer, neuronal loss in AD and PD diseases, pancreatic beta-cell depletion in T2D, podocyte death in MN, and osteoblast loss in IOP. These findings indicate that MTNR1A exerts a protective role against ferroptotic signaling. Mechanistic studies further reveal that MTNR1A knockdown enhances ferroptosis by suppressing the Sirt1/Nrf2/HO-1/GPX4 signaling axis and reducing Fth1 protein expression in SH-SY5Y cells (96). Whether this same mechanism occurs in other pathological conditions remains to be validated. Taken together, these data position MTNR1A not merely as a circadian regulator, but as a universal, cross-organ master switch essential for the inhibition of ferroptotic cell death.

Significant downregulation of MTNR1A across a spectrum of diseases (including MN, IOP, cancer, T2D, AD, and PD) raises the important question of whether targeting the restoration of MTNR1A levels offers a novel therapeutic strategy for these disorders. Previous studies have identified two compounds that can enhance MTNR1A levels. NG-497 (an adipose triglyceride lipase inhibitor) was found to increase MTNR1A protein levels, and 5-aza-2′-deoxycytidine (decitabine; a DNA methylation inhibitor) was shown to reactivate MTNR1A expression in oral cancer cells. However, NG-497 is currently a research-only compound with no investigational new drug status, no good laboratory practice toxicology data, and no clinical trials. Similarly, although decitabine (an FDA-approved DNA methylation inhibitor) is available, its use is restricted to cancer indications (myelodysplastic syndromes and acute myeloid leukemia) and thus cannot be applied for noncancer indications or for targeting MTNR1A promoter demethylation in a clinical setting. Given the increasing identification of diseases associated with lower MTNR1A levels, there is a critical need for new, clinically applicable drugs that can effectively enhance MTNR1A levels for therapeutic use.
